# SARS-CoV-2 viral variants can rapidly be identified for clinical decision making and population surveillance using a high-throughput digital droplet PCR assay

**DOI:** 10.1038/s41598-023-34188-7

**Published:** 2023-05-10

**Authors:** Olivier Pernet, Maia Weisenhaus, Chrysovalantis Stafylis, Christopher Williams, Mihaela Campan, Jonas Pettersson, Nicole Green, David M. Lee, Paul D. Thomas, Pamela Ward, Howard Hu, Jeffrey D. Klausner, Andrea A. Z. Kovacs, the U. S. C. Variant Study Group, Cassidy Hernandez-Tamayo, Sarah Van Orman, Frank Gilliland, David Conti, Angie Ghanem-Uzqueda, Daniel Yepez, Sofia Stellar, Aditya P. Tadanki, Jillian Max, Honour Fottrell, Ethan Ong, Sabrina Navarro, Kaelyn Moses, Michael Akaolisa, Bijan Hosseini, Shaleen Sunesara, Yuzhu Wang, Andrew Zaw, Earl Strum, Yolee Casagrande, Nathalie Hernandez-Rodriguez, Paul Thomas, Tara Chu, Jane Emerson

**Affiliations:** 1 Department of Pediatrics, Maternal, Child and Adolescent Center for Infectious Diseases and Virology, University of Southern California, Los Angeles, CA USA; 2 Department of Population and Public Health Sciences, University of Southern California, Los Angeles, CA USA; 3 Department of Preventive Medicine, Division of Bioinformatics, University of Southern California, Los Angeles, CA USA; 4 Department of Pathology & Laboratory Medicine in Keck, University of Southern California, Los Angeles, CA USA; 5 Los Angeles County Department of Public Health, Los Angeles, CA USA; 6 Department of Family Medicine, University of Southern California, Los Angeles, CA USA; 7 Keck Medicine, University of Southern California, Los Angeles, CA USA

**Keywords:** Infectious-disease diagnostics, SARS-CoV-2, Viral epidemiology

## Abstract

Epidemiologic surveillance of circulating SARS-CoV-2 variants is essential to assess impact on clinical outcomes and vaccine efficacy. Whole genome sequencing (WGS), the gold-standard to identify variants, requires significant infrastructure and expertise. We developed a digital droplet polymerase chain reaction (ddPCR) assay that can rapidly identify circulating variants of concern/interest (VOC/VOI) using variant-specific mutation combinations in the Spike gene. To validate the assay, 800 saliva samples known to be SARS-CoV-2 positive by RT-PCR were used. During the study (July 2020-March 2022) the assay was easily adaptable to identify not only existing circulating VAC/VOI, but all new variants as they evolved. The assay can discriminate nine variants (Alpha, Beta, Gamma, Delta, Eta, Epsilon, Lambda, Mu, and Omicron) and sub-lineages (Delta 417N, Omicron BA.1, BA.2). Sequence analyses confirmed variant type for 124/124 samples tested. This ddPCR assay is an inexpensive, sensitive, high-throughput assay that can easily be adapted as new variants are identified.

## Introduction

The Severe Acute Respiratory Syndrome Coronavirus 2 (SARS-CoV-2) has caused over 580 million infections globally and over 6.4 million deaths since its initial outbreak in 2019^1^. As the pandemic has progressed it has led to the emergence of novel viral lineages distinct from the original SARS-CoV-2. The World Health Organization (WHO) and Centers for Disease Control (CDC) monitor circulating variants worldwide and classify variants with increased transmissibility, disease severity or immune escape as "variants of concern" (VOC). They also monitor a set of "Variants of Interest” (VOI), which are variants with mutations associated with disease outcomes^2^. Thus far, there have been four major waves of infection: the first lineage to spread globally, B.1 (referred here as original sequence, Nextstrain clade 20A), followed by the VOCs Alpha, Delta, and now Omicron and its sub-lineages. Other VOCs have caused outbreaks in Brazil (Gamma) and South Africa (Beta) but did not reach worldwide prevalence. VOCs have hindered vaccine immunization efficacy and have led to increased re-infection in previously infected individuals^3^. It is expected that SARS-COV-2 will continually evolve and produce new variants if there is continued high level viral replication at the population level.

Whole genome sequencing (WGS) remains the gold standard for variant surveillance, as it can unambiguously identify known variants and detect new mutations and lineages as they arise. Sequencing-based methods, however, are costly, time consuming and require expertise and specialized equipment for analytical processing. As a result, availability of WGS is limited to specialized labs and cannot be implemented across all public health and clinical diagnostic laboratories. Further, the lack of sequencing availability in low-income countries hinders the ability to make public health decisions that are needed to manage the ongoing pandemic on a global scale and prevent future outbreaks^4^.

On the other hand, SARS-CoV-2 PCR-based assays are ubiquitous, as they serve as the gold standard for case identification. Several RT-PCR and ddPCR multiplex assays have been used to differentiate a limited number of variants^5–11^, or sub-lineages among these variants^10,12,13^. However, the increasing number of target sequences prevent the development of a comprehensive PCR-based assay that can differentiate a large number of variants and their sub-lineages based on variant-specific mutations.

As the World Health Organization continues to monitor a growing number of VOC and VOI, there is an urgent need for more globally accessible, rapid, and inexpensive diagnostic tools to identify current and new circulating variants. This is especially important not only for surveillance at the population level^4,14^, but also for decision making when it comes to prophylaxis and treatment with monoclonal antibodies and antivirals^15–17^.

Here we developed a PCR-based assay to rapidly identify mutations associated with most of the VOC and several sub-lineages. We field tested the protocol using 800 samples from the University of California (USC) Student Health and Keck Medicine of USC between July 23rd, 2020, and March 29th, 2022. Finally, we validated the ddPCR results with sequence analyses for a subset of 124 samples.

## Results

### Validation with standards samples

**Figure 1:**
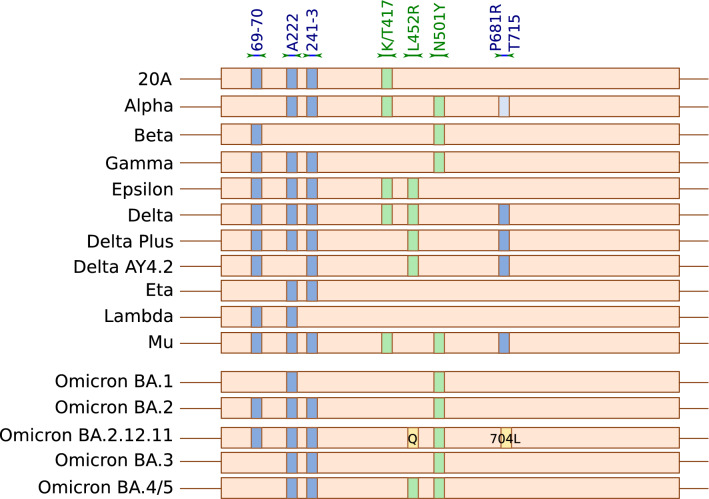
Primers/probe targets mapped onto the SARS-CoV-2 spike gene. Regions in blue indicate the targets of primers/probe sets using the FAM fluorophores, while regions in green indicate the targets of primers/probe sets using the HEX fluorophores.

**Figure 2:**
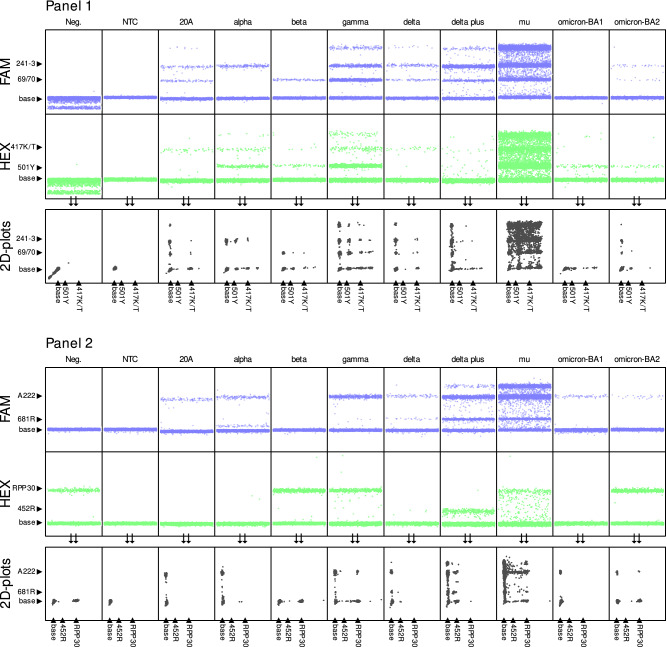
Identification of variants using control samples. Negative saliva, no-template control (NTC), and sequenced samples were analyzed by ddPCR. Results obtained in FAM (blue) and HEX (green) channel for panel 1 are presented on top figure panel, while panel 2 are presented on the lower figure panel. The combinations of HEX (x-axis) and FAM (y-axis) in the 2D-plots (gray) show the specific amplification pattern for each variant presented.

We tested our ddPCR protocol (see Methods and Fig. 1, Table 1, Supp. Figure 1 and 2, Supp. Table 1, 2, and 3) using a standard sample set that included: the Washington Isolate/20A (original), and the Alpha, Beta, Gamma, Delta, Delta K417N, Mu, Omicron BA.1, and Omicron BA.2 variants circulating during the study period (July 2020-March 2022). Variant determination was 100% accurate for controls with appropriate dilutions under our optimized cycling parameters (Fig. 2, Table 2). We also identified a unique pattern for the P681R-T715wt primer/probe set. While our assay detected the P681R, a hallmark of the Delta variants, it also consistently showed a unique, alternative pattern for the P681H/T715I found in the Alpha variant with a peak MFI higher than baseline but significantly lower than the 681R peak (Supp. Figure 3). Interestingly, the Mu variant, which has P681H like Alpha but lacks T715I, showed a signal comparable to P681R/T715wt (Supp. Table 4).

**Table 1:** Primers and probe target sequences.

Set	Forward sequence	Reverse sequence	Probe sequence	Probe fluorophore
69/70	CTGCATACACTAATTCTTTCAC	GGTCCCAGAGACATGTATAG	CAACTCAGGACTTGTTCTTACCTT	FAM
241–3	AATTTAGTGCGTGATCTCCC	AACTTCTATGTAAAGCAAGTAAAG	TTGCCAATAGGTATTAACATCACTAGGT	FAM
K/T417	CTTTTAAGTGTTATGGAGTGTT	CTGGTAATTTATAATTATAATCAGCAATC	CTCTGCTTTACTAATGTCTATGCAGATTCAT	HEX
501Y	GGTTTCCAACCCACTT	AGACTTTTTAGGTCCACAAAC	TTACCAACCATACAGAGTAGTAGTACTTTC	HEX
P681RT715wt	TCAGACTCAGACTAATTCTCG	CATAGACACTGGTAGAATTTCTG	CTCTATTGCCATACCCACAAATTTTAC	FAM
A222	AGGGTTTTTCGGCTTTAGAA	AACTTCTATGTAAAGCAAGTAAAG	AGGGTTTTTCGGCTTTAGAA	FAM
L452R	GTTGGTGGTAATTATAATTACCG	CATATGATTGTAAAGGAAAGTAACA	CCGGTAGCACACCTTGTAATG	HEX
RPP30	AGATTTGGACCTGCGAGCG	GAGCGGCTGTCTCCACAAGT	TTCTGACCTGAAGGCTCTGCGCG	HEX

**Table 2:**
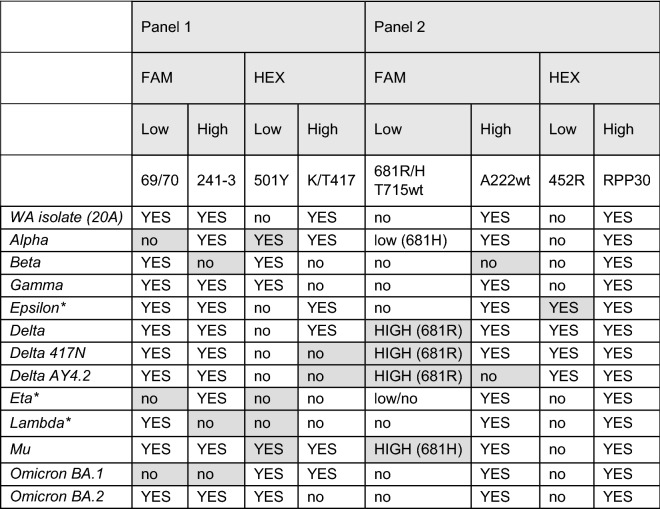
Specific signal pattern for each variant in the FAM and HEX channels. For each variant, targets that are expected to be amplified are noted by “YES”, and targets that are expected to not be amplified are marked by “no”. Fluorescent signals can be low or high based on probe concentrations. Variants with * indicate expected signals determined by in-silico analysis and were not confirmed with actual samples. Cells in grey indicate hallmark mutations required for identification.

### Discrimination between sub-lineages

In addition to the main circulating variants, it can be important to monitor for new sub-lineages within these variants. This was first highlighted by the emergence of the Delta Plus variant, and later by Omicron sub-lineages (BA.1, BA.2, BA.3, BA.4/5). Here we show that K/T417 primer/probe set allows the differentiation of the original Delta variant and the Delta K417N variant that carries the K417N mutation (Fig. 2). With the onset of Omicron, we also tested the ability of our assay to discriminate between Omicron BA.1 and BA.2. Omicron BA.1 yielded a unique amplification pattern (amplified only at viral targets A222, K/T417, N501Y). Omicron BA.2 however, amplified the same mutation profile as seen with Gamma (69/70, A222, 241–3, K/T417, N501Y) (Fig. 2). Thus, our assay could accurately discriminate Omicron BA.1 from the earlier VOCs and could distinguish BA.1 from BA.2 but cannot discriminate Gamma and BA.2.

### Determination of limit of identification using standard samples

We then proceeded to determine the limit of detection and limit of identification with the original sequence USA-WA1/2020. Standard, quantified samples from *BEI Resources* were spiked at different concentrations ranging from 2.5 to 2.5 × 10^6^ per reaction (3 × 10^-1^ copy/μL to 3 × 10^5^ copies/μL). USA-WA1/2020 sequence was identified in the sample at concentrations as low as 250 copies per reaction (Supp. Fig. 4, Supp. Table 5). Importantly, all 3 sequences required to identify the original sequence (69/70, 241–3, and A222) and 1 confirmation sequence (R/T417) were identifiable at 250 RNA copies/reaction.

Low counts (35 or less) of positive droplets for non-specific targets corresponding to low probe concentration (681R, 501Y, 452R) were observed with viral titers exceeding 25,000 RNA copies per reaction because of incomplete or sub-optimal amplification of the targets with higher probe concentration (“rain pattern” from A222, K/T417, and RPP30, respectively). However, even at high viral copy concentrations, the non-specific droplet number remained 100-fold (or more) lower than for the specific sequences and therefore did not interfere with data interpretation and variant identification, altogether, reliable fluorescent signals were achievable up to 250,000 copies per reaction.

### Analysis of remnant saliva samples from USC Student population and USC Keck Medicine population

The study was conducted as part of the ongoing COVID-19 testing programs for all students and staff. SARS-CoV-2 positive samples from *USC Student Health* and *USC Keck Medicine* (n = 800) were collected from July 23rd, 2020, through March 29th, 2022. When available, RT-PCR data provided by the *USC Department of Clinical Pathology* indicated Ct values for the S gene ranging from 12.49 to 32.99 (median = 20.925, mean = 21.18). Nucleic acids were isolated as described in the Methods section and analyzed with the two ddPCR panels. Results of variants detected are shown in Supp. Table 6 and 7. The original sequence was the dominant sequence in 2020 (250 sequences identified) and was replaced by Alpha (12 sequences) in the first semester of 2021, with Delta peaking in the third quarter of 2021 (255 sequences). After that Omicron was the dominant variant (278 sequences). Interestingly, we noticed that in samples with high viral loads, the K/T417 primer/probe set was able to weakly bind Omicron sequence (as seen in Supp. Figure 5), leading to a few positive droplets for that target, but at lower counts compared to the 501Y target in the same channel. Two Mu and two Gamma variants were detected in July 2021. Interestingly, one of the two Mu sequences only weakly amplified 417 T. In addition, we also identified 3 samples with unexpected patterns. Two of them were suspected to belong to the Delta lineage, as they carried the P681R and L452R, hallmarks of the delta lineage, which was confirmed by WGS (Supp. Table 7).

### WGS confirmed ddPCR analysis

We confirmed the ddPCR identification in a subset of 124 samples by Whole Genome Sequencing (WGS) and Single-Nucleotide Polymorphism (SNP) analysis, including 120 samples clearly identified by ddPCR and the 4 samples with an unexpected pattern. The subset of clearly identified samples included 13 samples with the original strain lineage, 9 Alpha, 72 Delta, 1 Mu, and 25 Omicron samples (Supp. Table 7). The four samples with non-VOC pattern included the atypical Mu (with weak T417 amplification) and the 2 Delta-like variants described above, as well as a sample that could not be clearly identified as any VOC.

As shown in Supp. Table 7, our ddPCR screening assays correctly identified all (120/120) samples subsequently sequenced. The mutation patterns observed by ddPCR was also confirmed for the 4 samples with non-VOC patterns. The Mu-like variant was confirmed, belonging to the Pango lineage B.1.6.21. Regarding the two Delta-like samples, the WGS confirmed the atypical pattern observed with ddPCR, and revealed that the first sample was from the Delta lineage, AY.2, which carries a substitution at spike amino acid position 70 (V70F) that impaired binding of the 69/70 primers-probe set. The second Delta-like sample was also confirmed as a Delta sub-lineage, related to the AY.44 lineage with a 69/70 deletion.

**Figure 3:**
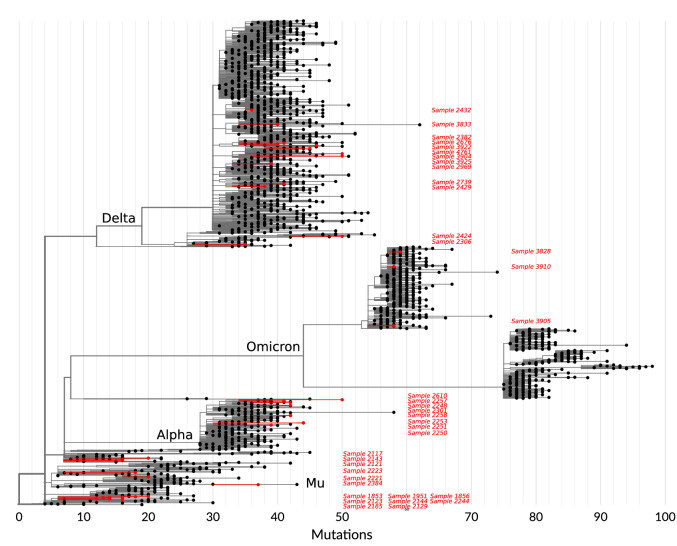
Phylogenetic tree with sequenced samples confirming ddPCR identification. Sequenced samples (red) were placed in SARS-CoV-2 phylogenetic tree built using UShER against the GISAID database containing 5 × 10^6^ sequences. A random down-sampling to 2,000 sequences (in black) was used to display the phylogenetic tree.

The last sample that could not be clearly linked to any VOC was determined to belong to the Pango Lineage B.1.1.207. Our ddPCR screening correctly determined wildtype sequences at position 69/70, 222, 241–3, 417, 452, 501. For this sample, our assay amplified the target P681R-T715wt with high MFI; the sequencing results determined that this sample indeed had the combination P681H and a wild type T715 (similar to the sequence in the Mu variant). Finally, 40 of the sequenced samples were mapped onto the phylogenetic tree shown in Fig. 3.

### Detection of multiple variants in mixed samples

**Figure 4:**
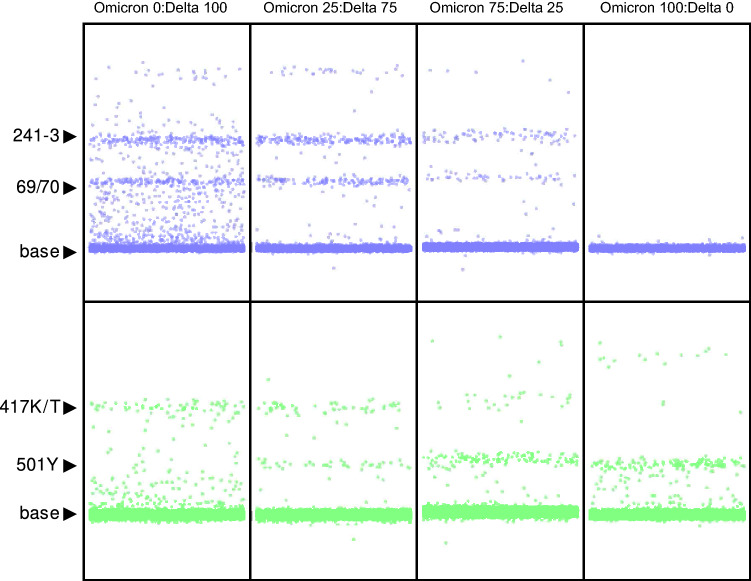
Multiple/mixed variant experiment. Negative saliva samples were spiked with different proportions of omicron BA.1 and Delta variants (0:100; 25:75; 75:25; and 100:0, from left to right), and the mixture was analyzed by ddPCR. Panel 1 confirmed a decrease of droplets in the Delta specific bands (69/70; 241–3, K/T417) with decreasing Delta concentrations, as well as an increase of Omicron BA-1 specific droplets (501Y) with increasing Omicron BA-1 concentrations.

Reports have described simultaneous co-infection by multiple variants in a single patient^18,19^. To mimic that situation, we mixed two control samples (Delta and BA.1) together at different ratios (0:100; 25:75; 75:25; and 100:0). Results for the mixing experiment indicated that the assay could differentiate between the two different conditions and does not confuse the mixture as a new variant (Fig. 4). As expected, when delta was the dominant variant in the mixture the Delta-specific targets (like P681R, 69/70, 241–3) were present in higher numbers and targets specific to BA.1 (501Y) were present in lower numbers.

## Discussion

With the continued evolution of SARS-CoV-2 variants it is critical to have a more rapid and widely available method to detect evolving variants. This is essential not only to monitor trends at the population level, but also to assess for specific variants that may impact clinical decision making related to use of antivirals and monoclonal antibodies for patients. To this end, we developed a sensitive and specific ddPCR assay for the discrimination of current VOC and VOI.

To our knowledge, our ddPCR assay can screen the largest number of SARS-CoV-2 spike gene mutations in the fewest reactions^5–7,10,12,20^. The variants Alpha (B.1.1.7, Q.1-Q.8), Beta (B.1.351, B.1.351.2, B.1.351.3), Gamma (P.1, P.1.1, P.1.2), Delta (B.1.617.2 and sub-lineages), Mu (B1.621), Eta (B.1.525), Lamda (C.37), Omicron (B1.1.529 and sub-lineages), and the original strain (B.1) were all identifiable based on their unique pattern of mutations.

Over the 20 months of this study our assay evaluated 800 remnant saliva samples that were previously tested positive for COVID-19 by RT-PCR. We were able to detect and identify relevant new variants as they emerged and a subset of which was further confirmed by WGS. The assay now targets seven spike protein mutations to qualitatively discriminate between the original strain, VOC/VOI, and even the specific sub-lineages Delta Plus and Omicron BA.1 and BA.2 and also should be able to differentiate the BA.3 and BA.4/5 variants^12,17^.

The lower limit of RNA input required for unambiguous variant identification was 250 copies per reaction, and reactions with as much as 250,000 RNA copies were identifiable. Thus, our screening method is effective with RNA loads across three orders of magnitude and compatible with clinical use^21^. Assay sensitivity may be increased even further by separating the 1-step RT-ddPCR into a reverse transcription reaction and a ddPCR reaction, as was demonstrated by *Telwatte* *et al.* ^22^.

The current gold standard for variant surveillance efforts is WGS. However, WGS is still an expensive technology and is not available outside major research/surveillance centers. As an alternative to sequencing, several groups have developed PCR-based assays for SARS-CoV-2 variant identification. Indeed, identifying variants requires the simultaneous evaluation of many shared genetic mutations which can easily be done with multiplex PCR methods. Furthermore, the targets of our assay can be removed, substituted, or modified as the pandemic progresses, and new mutations become relevant. For example, the K/T417 set was added to differentiate between Delta and Delta K417N and could now be replaced to characterize future Omicron sub-lineages. Importantly, unlike WGS, our PCR based assay allows for variant determination in hours. Further, there may be limitations in using WGS^23^ to identify low level circulation of minor variants in the background of dominant variants such as may occur during waste-water monitoring, or with co-infection by two distinct variants. We demonstrated that our assay was able to identify a combination of variants within a single sample.

To demonstrate the use of our assay in the field we classified variants circulating within the *USC Student* and *USC Keck Medicine* community between Fall of 2020 and Spring 2022. Consistent with Los Angeles County reporting, most samples from 2020 were the original sequence, with Alpha gaining prevalence in April 2021. As expected, Delta infections were observed as early as June 2021 and became dominant in July 2021. Finally, Omicron displaced Alpha and Delta as the dominant variant by the end of December 2021. Thus, we were able to characterize four different waves of dominant variants. Importantly, we were able to identify sub-lineages such as Delta K417N and Omicron BA.1/BA.2. Unique to our study is that we identified new VOC as they emerged and evolved over an almost two-year study period.

In addition to the four most common variants, we detected four more viral lineages: Gamma, Mu, Delta sub-lineage AY.2, and B.1.1.207. The two Mu cases were from July 2021, which was during the peak of Mu cases in the USA^24,25^. While our ddPCR assay was not designed for discrimination of B.1.1.207 or AY.2, it did detect their unique mutation patterns. The B.1.1.207 sample was collected from October 2020, just months after the identification of this lineage in Nigeria^26^. To date, there are only 57 B.1.1.207 genomes from California^27^. In contrast, the AY.2 lineage was most prevalent in the USA, with more than half of the cases (1,540 of 2940 global) localized to California. The lack of amplification for 69/70 can be explained by a V70F substitution, which occurred in AY.2 but not in other Delta lineages^28^.

The key to discriminating variants is that each variant needs to elicit a unique amplification pattern. One drawback of this assay is that the mutation pattern we classified as Delta (wild type for 69/70, 222, 241–243, K417 and N501, and substitutions L425R, P681R) is the same as we would expect for the rare variant, Kappa. We do not expect that many of our Delta classified samples to include Kappa, however, due to the small number of Kappa cases reported in California (92 total) or the USA at large (341 total). Groups that are concerned with differentiating these two lineages could add an additional PCR target to the assay, such as S-gene E156G, T19R, T478K, E484Q, D950N or Q1071H. Our assay also yielded the same amplification pattern for Omicron BA.2 and Gamma. These two variants never overlapped temporally, making them differentiable by date. PCR targets could also be added or modified to discriminate the two in relevant settings.

While our assay evaluates a broad range of spike gene targets, it was designed to assess either the wild type or mutated sequences only. Therefore, the lack of amplification is used to make inferences of a non-amplified sequence. The dropout of certain PCR targets has been the basis for monitoring of variant distributions at a global scale^29–33^. To unambiguously assess mutated versus wildtype sequences, one could include separate primer/probe sets for wild type and mutated sequences, as has been done previously by others^5,20^. The trade-off to that approach is that it requires increasing the number of PCR reactions overtime or reducing the number of viral targets^11^. In contrast, melting curve assays, work by amplifying all targets and discriminating wild type sequences from those mutated based on the temperatures at which they denature ^34,35^. While melting curve assays allow for fewer primer/probe sets, reactions are limited to a small number of targets, and sequences with similar melting temperatures can be difficult to distinguish.

This assay has the potential to broadly expand capabilities to detect and monitor for changes in variant type at the population level utilizing this simple assay that bypasses WGS. Because it relies on PCR, this assay can be adapted to different settings such as large centers processing hundreds of samples. Also, the assay can be transferred to a fully hands-off, high-throughput system such as QXOne (Bio-Rad) that can perform the two panels in one reaction. Because of the binary nature of the ddPCR read out, the results can be easily analyzed by an automated script. This would be a valuable tool for large surveillance programs that currently rely on sequencing, providing a faster alternative. The assay can also be “downgraded” to a simpler multiplex RT-PCR. RT-PCR has become the gold standard worldwide for COVID-19 testing. The equipment is already available in most clinical laboratories and local surveillance centers.

Our PCR test could be implemented for fast and cost-efficient variant screening. This more rapid method can be easily utilized in the clinical setting to assess for known mutations that impact vaccine efficacy and response to treatment or prophylaxis with monoclonal antibodies and antivirals. Finally, the ability to monitor mixed variants makes it an ideal strategy for early detection of variant emergence. For example, it is well suited to detect a minority variant in waste-water surveillance sentinel system.

## Methods

### Overall study design

This study included 800 de-identified remnant saliva samples collected during the USC SARS-CoV-2 infection monitoring program. Participants included *USC Student Health* populations and *Keck Medicine at USC* students, faculty, and employees that tested positive for SARS-CoV-2 by RT-PCR. Participants provided their written informed consent. The study is approved by the University of Southern California Institutional Review Board IRB# HS-21–00,366, IRB# UP-21–00,393. All methods were performed in accordance with the relevant guidelines and regulations.

Saliva was obtained using a *s*aliva-chew method. Individuals chewed on a swab until approximately 2 mL of saliva was collected in a vacuette tube without additives. Samples were transferred to the USC Clinical Pathology Laboratory within 2 h of collection. Upon receipt, samples were heat-inactivated at 65 °C prior to SARS-CoV-2 testing with RT-PCR. Positive remnant samples were stored at –80C.

### Total nucleic acid extraction

The Magbind Viral DNA/RNA 96 Kit (M62646, Omega Bio-Tek) was used to extract total nucleic acids from patient samples following the manufacturers protocol. Extraction was done on either the KingFisher Apex Purification System (Cat#5,400,930, ThermoScientific) or KingFisher Duo Prime Purification System (Cat#5,400,110, ThermoScientific). Samples were eluted into 75μL of nuclease-free water and stored at -80℃. A QIAamp Viral RNA Kit was used to extract RNA from a subset of samples that were sent for genomic sequencing. These samples were eluted into 60μL nuclease free water and nucleic acids were quantified with a ThermoScientific Nanodrop One C (Cat#ND-ONEC-W, ThermoScientific).

### Primer design

We designed seven primer/probe sets targeting hallmark mutations in the SARS-CoV-2 Spike protein gene that would enable characterization of VOCs. Primers were made to amplify wild type or mutated sequences at S-gene amino acid positions 69/70, 222, 241–3, K417 and K417T, L452R, N501Y and P681R. Additionally, an internal control set of primers targeting the human RNase P / Protein Coding Gene 30 (RPP30) was included, using the sequences previously published by the US CDC (Centers for Disease Control) as an internal control for SARS-CoV-2 (CDC#2019-nCoV EUA-01). Sequences are described in Table 1 and mapped in Fig. 1. All probes were modified with 3’BHQ and carried on their 5’ end either HEX or FAM (Table 1). Primer–probe sets were divided into two ddPCR panels, each combining 2 FAM- and 2 HEX-labeled sets as described in Supp. Table 1 and 2. PCR panel 1 primers amplified amino acid sites 69/70, 241–243, K417 wild type and K417T (but not K417N), and N501Y. Panel 2 amplified L452R, P681R/H-T715wt (jointly), and the human DNA control, RPP30. A set targeting A222 was subsequently added to panel 2 as an internal control for viral genome. All primers were used at a concentration of 0.100 μM, with FAM and HEX probes at 0.100, 0.200, or 0.300 μM concentrations (as described in Supp Table 1) so that each target elicited a unique fluorescence intensity.

### Establishing optimal temperatures for RT and PCR steps of the ddPCR

Optimal temperatures for the RT step for each panel were established using a 52 to 58℃ temperature gradient. PCR was performed according to manufacturer recommendations. Results showed an optimal RT temperature of 54.5℃ for both panel 1 and 2 (Supp. Figure 1). A second PCR gradient was used to determine the optimal PCR annealing temperatures. Results show an optimal annealing temperature of 55℃ for panel 1 and 56℃ for panel 2, respectively (Supp. Figure 2).

### Digital droplet PCR

Purified total nucleic acid extractions were used for viral genome amplification by digital droplet ddPCR. One-Step RT ddPCR Advanced Kit for Probes (Bio-Rad #1,864,022) were combined with primers and probes to create a PCR master mix. 70 μL of emulsified droplets were generated on a QX Droplet Generator (Bio-Rad) from 20 μL reaction volumes, which were then loaded onto a Bio-Rad CFX96 Deep Well Real-Time thermocycler using the cycle parameters detailed in Supp. Table 3. Droplet fluorescence was measured using a QX200 Droplet Reader (#1,864,003, Bio-Rad) paired with QuantaSoft software (version 1.7.4, Bio-Rad). Each run included control samples for Alpha, Delta, Delta K417N (Supp. Table 2) spiked in a saliva sample from an uninfected individual, and a no-template control (nuclease free water). Highly concentrated samples were diluted prior to ddPCR to avoid saturation of the assay and limit sub optimal amplification in droplets (“rain pattern”). To be considered positive for the mutation, a positive band required 5 droplets at the expected intensity (excluding droplets from “rain pattern”). Samples with 1 to 5 droplets at any expected intensity were repeated.

We designed two multiplex ddPCR reactions that target a total of seven viral sequences which allow the discrimination between the variants Alpha, Beta, Gamma, Delta, Delta K417N, Lambda, Mu, Omicron BA.1 and BA.2, and the original sequence (USA-WA1/2020, “Washington Isolate”, NextStrain Clade 20A). Purified total viral nucleic acids were analyzed by ddPCR using a Bio-Rad QX200 system. Data was analyzed with QuantaSoft software (version 1.7.4, Bio-Rad) and a custom-made R pipeline (R version 4.0.5, R Foundation for Statistical Computing). Results and variant identification were independently reviewed and confirmed by 3 investigators (OP, MW, AK).

### Standards for validation and inactivated virus sources

Heat inactivated viruses of the Washington Isolate USA-WA1/2020 (NR-52286), Alpha (NR-55245), Beta (NR-55350), Delta (NR-56128), and Omicron (NR-56495) variants were obtained from NIH’s Biodefense and Emerging Infections Research Resources Repository (*BEI Resources*). Additionally, remnant saliva samples from patients infected with Gamma, Delta, Delta K714N and Omicron variants that were heat-inactivated, sequenced, and stored at –80℃ in Zymo DNA/RNA Shield (Zymo) were provided by *Curative Inc* (San Dimas, CA).

### Whole genome sequencing

A total of 124 samples were submitted for whole-genome sequencing, including 10 at *UCLA Technology Center for Genomics and Bioinformatics* (TCGB), 31 to *Azenta Life Science,* and 83 to the USC *Department of Pathology and Clinical Medicine* in collaboration with the *Los Angeles Department of Public Health* (LAC DoPH). All the samples were prepared using Illumina’s COVIDSeq Test kit (*Cat#20,043,675, Illumina*) and Illumina PCR Indexes (*Cat*#20,043,137, Illumina). All samples underwent RNA to cDNA conversion, index tagging, and PCR amplification as a part of library preparation. Sequencing was completed on an Illumina MiSeq Sequencer using a 300 cycle MiSeq Reagent Kit (Cat#MS-102–2002, Illumina). RNA-seq libraries were constructed using Stranded RNA-Seq Library Preparation Kit (Cat#KK8400, Kapa Biosystems), and sequenced using an Illumina HiSeq 3000 sequencer.

### Whole genome analysis

FASTQ files were assembled via alignment to the SARS-CoV-2 reference genome using Illumina's DRAGEN Covid Lineage application to generate a consensus sequence. Sequence reads were mapped into the global phylogenetic tree and assignment of the Pango lineage was performed by Ultrafast Sample placement on Existing tRee (UshER)^36^. Down-sampled global tree was visualized using Nextrain.org website^37,38^ and colors were edited using Inkscape v1.1 (*The Inkscape Project*).

### Conference presentation

Portions of this work were recently presented during the Negative Strand Virus Conference 2022 (NSV-2022) in Braga, Portugal, and during the International Conference of Emerging Infectious Diseases 2022 (ICEID-2022) in Atlanta, USA.

## Supplementary Information

Supplementary Information.

## Data Availability

The WGS datasets generated by USC *Department of Pathology and Clinical Medicine and* by the *Los Angeles County Department of Public Health,* and analysed during the current study are available in the *Global Initiative on Sharing Avian Influenza Data* repository (GISAID.org), accession numbers EPI_ISL_15550022 to EPI_ISL_15550082, EPI_ISL_15573087, and EPI_ISL_3299042. The WGS datasets generated by *UCLA TGCB* and *Azenta* and analysed during the current study are available in *GenBank* repository (ncbi.nlm.nih.gov), accession numbers OP752451 to OP752482.
