# Supplementary material for: SARS-CoV-2 viral variants can rapidly be identified for clinical decision making and population surveillance using a high-throughput digital droplet PCR assay
Source: Sci Rep. 2023 May 10;13:7612. doi: 10.1038/s41598-023-34188-7 (PMC10170421; doi:10.1038/s41598-023-34188-7)
Supplement: Supplementary file 1 — Supplementary Information. [file 41598_2023_34188_MOESM1_ESM.docx]

Supplementary material

**SUPPLEMENTARY TABLES**

|  |  | FAM  (low concentration) | FAM  **(high concentration)** | HEX  (low concentration) | HEX  (high concentration) |
| --- | --- | --- | --- | --- | --- |
| **Panel 1** | *Target*  *Probe Concentration*  *Exp. Intensity Units* | 69/70  100nM  (5000-6200 IU) | 241-3  200nM  (7100-8500 IU) | N501Y  200nM  (2600-3800 IU) | 417K/T  300nM  (5300-6400 IU) |
| **Panel 2** | *Target*  *Probe Concentration*  *Exp. Intensity Units* | P681R  100nM  *(4000-5000 IU)* | *A222*  *200nM*  *(7000-9000)* | L452R  100nM  (1850-2800 IU) | RPP30  200nM  (3100-5000 IU) |

***Supp Table1: Panel 1 and 2 descriptions:*** *each panel consists of two targets in the HEX channel and two targets in the FAM channel. Within each channel one target had a low concentration of fluorophore [0.100] or [0.200] µM and the other target had a high concentration of fluorophore, [0.200] or [0.300] µM. Fluorescent intensity is shown under each target.*

|  | ***69/70*** | ***241-3*** | ***417K/T*** | ***452*** | ***N501Y*** | ***P681R***  ***T715wt*** | ***A222V*** |
| --- | --- | --- | --- | --- | --- | --- | --- |
| *Positive control* | WA,δ, δ+ | WA,α,δ, δ+ | WA,α,δ | WA,α | α | δ, δ+ | WA, α, δ, δ+ |
| *Negative control* | α |  | δ+ | δ,δ+ | WA, δ, δ+ | WA |  |

***Supp. Table 2****: Variants used as positive and negative controls for each target sequence. WA: Washington Isolate, α: alpha variant, ,δ: delta variant, ,δ+: delta variant K417N*

|  | **Temperature (℃ )** | **Time**  **(minutes:seconds)** | **Repeat** |
| --- | --- | --- | --- |
| **Hold** | 25 | 02:00 |  |
| **Reverse Transcription** | 54.5 | 10:00 |  |
| **RT Inactivation** | 95 | 10:00 |  |
| **Denaturation**  **Annealing/Extension** | 95  55 (Panel 1)  56 (Panel 2) | 00:03  00:30 | 54x |
| **Enzyme Deactivation** | 98 | 10:00 |  |
| **Hold** | 12 | ∞ |  |

**Supp. Table 3:** RT-ddPCR thermocycling protocol

|  | P681 | T715 | Fluo. intensity |
| --- | --- | --- | --- |
| **20A- WA isolate** | C | C | baseline |
| **Alpha** | C -> A | C-> T | low |
| **Delta** | C -> G | C | high |
| **Mu** | C -> A | C | high |
| **20B** | C -> A | C | high |

***Supp.Table 4: Primers-probe set P681R/T715wt.*** *P681R/T715wt* *allows discrimination of Alpha variants due to their unique sequence. Polymorphisms/substitutions at P681 and T715 produce a uniquely low Fluorescent Intensity for Alpha compared to Delta, Mu, or the original strain.*

| Copies per reaction | **69/70** | **241-3** | *N501Y* | **K/T417** | *m681r* | *m681l* | **A222V** | *L452R* |
| --- | --- | --- | --- | --- | --- | --- | --- | --- |
| 2,500,000 | **5550** | **5900** | *0* | **0** | *29* | *NA* | **9495** | *14* |
| 2,500,000 | **3980** | **4089** | *0* | **0** | *19* | *NA* | **8150** | *31* |
| 250,000 | **2798** | **3389** | *27* | **1161** | *16* | *NA* | **2597** | *2* |
| 250,000 | **2807** | **3544** | *32* | **1170** | *15* | *NA* | **2756** | *1* |
| 25,000 | **265** | **364** | *2* | **130** | *1* | *NA* | **278** | *1* |
| 25,000 | **275** | **364** | *3* | **110** | *0* | *NA* | **257** | *0* |
| 2,500 | **21** | **43** | *0* | **8** | *0* | *NA* | **40** | *0* |
| 2,500 | **38** | **41** | *0* | **11** | *0* | *NA* | **28** | *0* |
| 250 | **0** | **2** | *0* | **1** | *0* | *NA* | **3** | *0* |
| 250 | **1** | **3** | *0* | **2** | *0* | *NA* | **3** | *0* |
| 25 | **0** | **0** | *0* | **0** | *0* | *NA* | **0** | *0* |
| 25 | **0** | **0** | *0* | **0** | *0* | *NA* | **0** | *0* |
| 2.5 | **0** | **0** | *0* | **0** | *0* | *NA* | **0** | *0* |
| 2.5 | **0** | **0** | *0* | **0** | *0* | *NA* | **0** | *0* |
| 0 | **0** | **0** | *0* | **0** | *0* | *NA* | **0** | *0* |
| 0 | **0** | **0** | *0* | **0** | *0* | *NA* | **0** | *0* |

***Supp. Table 5****: Total positive droplet numbers for each target at different concentrations of WA isolate. Columns in bold font indicate targets expected to be found in the WA isolate. Columns in italic font indicate targets expected to be absent in the WA isolate.*

| **Variant** | **Count** |
| --- | --- |
| 20A | 250 |
| Alpha | 12 |
| Gamma | 2 |
| Delta | 251 |
| Delta K417N | 4 |
| Mu | 2 |
| Omicron BA.1 | 277 |
| Omicron BA.2 | 1 |
| B.1.1.207 | 1 |
| *Total* | *800* |

***Supp. Table 6: Variants detected by ddPCR*** *in samples collected from July 23^rd^, 2020, and March 29^th^ 2022.*

| ***Date of collection*** | ***Reference*** | ***Sequenced by*** | ***Sequencing identification*** | ***ddPCR*** ***identification*** |
| --- | --- | --- | --- | --- |
| *NA* | *Delta* | *Curative* | *Delta* | *Delta* |
| *NA* | *Delta F9* | *Curative* | *Delta 417N* | *Delta 417N* |
| *NA* | *Alpha* | *Curative* | *Alpha* | *Alpha* |
| *NA* | *Gamma* | *Curative* | *Gamma* | *Gamma* |
| *2020-08-31* | *1853* | *UCLA TCGB* | *20A* | *original* |
| *2020-08-31* | *1856* | *UCLA TCGB* | *20A* | *original* |
| *2020-09-08* | *1951* | *UCLA TCGB* | *20A* | *original* |
| *2020-10-01* | *2117* | *UCLA TCGB* | *B.1.1207* | *Non VoC* |
| *2020-10-03* | *2121* | *Azenta* | *20B* | *original* |
| *2020-10-03* | *2123* | *Azenta* | *20A* | *original* |
| *2020-10-04* | *2129* | *Azenta* | *20A* | *original* |
| *2020-10-05* | *2143* | *Azenta* | *20B* | *original* |
| *2020-10-05* | *2144* | *Azenta* | *20A* | *original* |
| *2020-09-26* | *2165* | *Azenta* | *20A* | *original* |
| *2020-10-08* | *2212* | *Azenta* | *20A* | *original* |
| *2020-10-08* | *2221* | *Azenta* | *20G* | *original* |
| *2020-10-08* | *2223* | *Azenta* | *20C* | *original* |
| *2020-10-08* | *2225* | *Azenta* | *20A* | *original* |
| *2021-04-16* | *2248* | *Azenta* | *Alpha* | *Alpha* |
| *2021-04-16* | *2250* | *Azenta* | *Alpha* | *Alpha* |
| *2021-04-16* | *2251* | *Azenta* | *Alpha* | *Alpha* |
| *2021-04-23* | *2253* | *Azenta* | *Alpha* | *Alpha* |
| *2021-04-23* | *2257* | *Azenta* | *Alpha* | *Alpha* |
| *2021-04-30* | *2258* | *Azenta* | *Alpha* | *Alpha* |
| *2021-05-04* | *2263* | *UCLA TGCB* | *Alpha* | *Alpha* |
| *2021-06-23* | *2301* | *Azenta* | *Alpha* | *Alpha* |
| *2021-06-25* | *2306* | *UCLA TCGB* | *Delta (AY.2 / 21I)* | *Delta-like (atypical)* |
| *2021-08-01* | *2610* | *UCLA TCGB* | *Alpha* | *Alpha* |
| *2021-08-11* | *2739* | *UCLA TCGB* | *Delta* | *Delta* |
| *2021-07-09* | *2331* | *Azenta* | *Mu* | *Mu-like* |
| *2021-07-14* | *2384* | *UCLA TCGB* | *Mu* | *Mu* |
| *2021-07-14* | *2382* | *UCLA TCGB* | *Delta (AY.44)* | *Delta* |
| *2021-07-20* | *2424* | *Azenta* | *Delta* | *Delta* |
| *2021-07-20* | *2429* | *Azenta* | *Delta* | *Delta* |
| *2021-07-21* | *2432* | *Azenta* | *Delta* | *Delta* |
| *2021-08-04* | *2969* | *Azenta* | *Delta (AY.43)* | *Delta* |
| *2021-08-31* | *3116* | *USC Path.&Lab.Med.* | *Delta (AY .100)* | *Delta* |
| *2021-08-31* | *3118* | *USC Path.&Lab.Med.* | *Delta (AY .20)* | *Delta* |
| *2021-09-01* | *3146* | *USC Path.&Lab.Med.* | *Delta (AY .25)* | *Delta* |
| *2021-09-02* | *3165* | *USC Path.&Lab.Med.* | *Delta (AY .25)* | *Delta* |
| *2021-09-03* | *3188* | *USC Path.&Lab.Med.* | *Delta (AY .20)* | *Delta* |
| *2021-09-04* | *3191* | *USC Path.&Lab.Med.* | *Delta (AY .44)* | *Delta* |
| *2021-09-05* | *3196* | *USC Path.&Lab.Med.* | *Delta (AY .103)* | *Delta* |
| *2021-09-07* | *3218* | *USC Path.&Lab.Med.* | *Delta (AY .26)* | *Delta* |
| *2021-09-07* | *3220* | *USC Path.&Lab.Med.* | *Delta (AY .44* | *Delta* |
| *2021-09-07* | *3221* | *USC Path.&Lab.Med.* | *Delta (AY .25)* | *Delta* |
| *2021-09-10* | *3258* | *USC Path.&Lab.Med.* | *Delta (AY .48)* | *Delta* |
| *2021-09-10* | *3259* | *USC Path.&Lab.Med.* | *Delta (AY .48)* | *Delta* |
| *2021-09-10* | *3260* | *USC Path.&Lab.Med.* | *Delta (AY .103)* | *Delta* |
| *2021-09-10* | *3261* | *USC Path.&Lab.Med.* | *Delta (AY .103)* | *Delta* |
| *2021-09-11* | *3262* | *USC Path.&Lab.Med.* | *Delta (AY .116.1)* | *Delta* |
| *2021-09-11* | *3265* | *USC Path.&Lab.Med.* | *Delta (AY .103)* | *Delta* |
| *2021-09-13* | *3279* | *USC Path.&Lab.Med.* | *Delta (AY .119)* | *Delta* |
| *2021-09-15* | *3300* | *USC Path.&Lab.Med.* | *Delta (B.1.617.2)* | *Delta* |
| *2021-09-15* | *3301* | *USC Path.&Lab.Med.* | *Delta (AY .25)* | *Delta* |
| *2021-09-16* | *3311* | *USC Path.&Lab.Med.* | *Delta (AY .25* | *Delta* |
| *2021-09-17* | *3326* | *USC Path.&Lab.Med.* | *Delta (B.1.617.2)* | *Delta* |
| *2021-09-19* | *3332* | *USC Path.&Lab.Med.* | *Delta (AY .118)* | *Delta* |
| *2021-09-20* | *3333* | *USC Path.&Lab.Med.* | *Delta (AY .25)* | *Delta* |
| *2021-09-21* | *3339* | *USC Path.&Lab.Med.* | *Delta (AY .25.1)* | *Delta* |
| *2021-09-27* | *3379* | *USC Path.&Lab.Med.* | *Delta (AY .3)* | *Delta* |
| *2021-09-27* | *3380* | *USC Path.&Lab.Med.* | *Delta (AY .47)* | *Delta* |
| *2021-09-29* | *3394* | *USC Path.&Lab.Med.* | *Delta (AY .44)* | *Delta* |
| *2021-10-01* | *3405* | *USC Path.&Lab.Med.* | *Delta (AY .44)* | *Delta* |
| *2021-10-01* | *3406* | *USC Path.&Lab.Med.* | *Delta (AY .25)* | *Delta* |
| *2021-10-02* | *3409* | *USC Path.&Lab.Med.* | *Delta (AY .44)* | *Delta* |
| *2021-10-03* | *3412* | *USC Path.&Lab.Med.* | *Delta (AY .4)* | *Delta* |
| *2021-10-04* | *3413* | *USC Path.&Lab.Med.* | *Delta (AY .122)* | *Delta* |
| *2021-10-06* | *3425* | *USC Path.&Lab.Med.* | *Delta (AY .103)* | *Delta* |
| *2021-10-07* | *3430* | *USC Path.&Lab.Med.* | *Delta (AY .44)* | *Delta* |
| *2021-10-08* | *3441* | *USC Path.&Lab.Med.* | *Delta (AY .103)* | *Delta* |
| *2021-10-08* | *3443* | *USC Path.&Lab.Med.* | *Delta (AY .39)* | *Delta* |
| *2021-10-12* | *3452* | *USC Path.&Lab.Med.* | *Delta (AY .3)* | *Delta* |
| *2021-10-12* | *3454* | *USC Path.&Lab.Med.* | *Delta (AY .44)* | *Delta* |
| *2021-10-13* | *3455* | *USC Path.&Lab.Med.* | *Delta (AY .100)* | *Delta* |
| *2021-10-13* | *3456* | *USC Path.&Lab.Med.* | *Delta (AY .25)* | *Delta* |
| *2021-10-13* | *3457* | *USC Path.&Lab.Med.* | *Delta (AY .44)* | *Delta* |
| *2021-10-18* | *3477* | *USC Path.&Lab.Med.* | *Delta (AY .100)* | *Delta* |
| *2021-10-18* | *3479* | *USC Path.&Lab.Med.* | *Delta (AY .3)* | *Delta* |
| *2021-10-18* | *3480* | *USC Path.&Lab.Med.* | *Delta (AY .100)* | *Delta* |
| *2021-10-18* | *3481* | *USC Path.&Lab.Med.* | *Delta (AY .100)* | *Delta* |
| *2021-10-18* | *3484* | *USC Path.&Lab.Med.* | *Delta (AY .44)* | *Delta* |
| *2021-10-21* | *3506* | *USC Path.&Lab.Med.* | *Delta (AY .26)* | *Delta* |
| *2021-10-25* | *3513* | *USC Path.&Lab.Med.* | *Delta (AY .3)* | *Delta* |
| *2021-10-25* | *3514* | *USC Path.&Lab.Med.* | *Delta (AY .100)* | *Delta* |
| *2021-10-25* | *3515* | *USC Path.&Lab.Med.* | *Delta (AY .103)* | *Delta* |
| *2021-10-27* | *3537* | *USC Path.&Lab.Med.* | *Delta (AY .44)* | *Delta* |
| *2021-10-27* | *3540* | *USC Path.&Lab.Med.* | *Delta (AY .44)* | *Delta* |
| *2021-10-28* | *3544* | *USC Path.&Lab.Med.* | *Delta (AY .3)* | *Delta* |
| *2021-10-28* | *3545* | *USC Path.&Lab.Med.* | *Delta (AY .3)* | *Delta* |
| *2021-10-28* | *3546* | *USC Path.&Lab.Med.* | *Delta (AY .3)* | *Delta* |
| *2021-10-29* | *3558* | *USC Path.&Lab.Med.* | *Delta (AY .126)* | *Delta* |
| *2021-10-29* | *3559* | *USC Path.&Lab.Med.* | *Delta (AY .100)* | *Delta* |
| *2021-10-30* | *3561* | *USC Path.&Lab.Med.* | *Delta (AY .25.1)* | *Delta* |
| *2021-11-04* | *3586* | *USC Path.&Lab.Med.* | *Delta (AY .44)* | *Delta* |
| *2021-11-05* | *3607* | *USC Path.&Lab.Med.* | *Delta (AY .44)* | *Delta* |
| *2021-11-06* | *3608* | *USC Path.&Lab.Med.* | *Delta (AY .103)* | *Delta* |
| *2021-11-12* | *3676* | *Azenta and USC Path.&Lab.Med.* | *Delta (AY .44)* | *Delta-like (atypical)* |
| *2021-12-14* | *3828* | *Azenta* | *Omicron BA.1* | *Omicron BA.1* |
| *2021-12-15* | *3833* | *Azenta* | *Delta* | *Delta* |
| *2021-12-18* | *3904* | *Azenta* | *Delta (AY .3)* | *Delta* |
| *2021-12-19* | *3905* | *Azenta* | *Omicron BA.1* | *Omicron BA.1* |
| *2021-12-20* | *3910* | *Azenta* | *Omicron BA.1* | *Omicron BA.1* |
| *2021-12-20* | *3922* | *Azenta* | *Delta (AY .44)* | *Delta* |
| *2021-12-20* | *3925* | *Azenta* | *Delta (AY .3)* | *Delta* |
| *2022-01-04* | *4761* | *Azenta* | *Delta (AY .3)* | *Delta* |
| *2022-02-03* | *6001* | *USC Path.&Lab.Med.* | *Omicron BA.1* | *Omicron BA.1* |
| *2022-02-03* | *6007* | *USC Path.&Lab.Med.* | *Omicron BA.1* | *Omicron BA.1* |
| *2022-02-03* | *6008* | *USC Path.&Lab.Med.* | *Omicron BA.1* | *Omicron BA.1* |
| *2022-02-05* | *6009* | *USC Path.&Lab.Med.* | *Omicron BA.1* | *Omicron BA.1* |
| *2022-02-05* | *6010* | *USC Path.&Lab.Med.* | *Omicron BA.1* | *Omicron BA.1* |
| *2022-02-05* | *6014* | *USC Path.&Lab.Med.* | *Omicron BA.1* | *Omicron BA.1* |
| *2022-02-04* | *6015* | *USC Path.&Lab.Med.* | *Omicron BA.1* | *Omicron BA.1* |
| *2022-02-06* | *6020* | *USC Path.&Lab.Med.* | *Omicron BA.1* | *Omicron BA.1* |
| *2022-02-07* | *6027* | *USC Path.&Lab.Med.* | *Omicron BA.1* | *Omicron BA.1* |
| *2022-02-07* | *6034* | *USC Path.&Lab.Med.* | *Omicron BA.1* | *Omicron BA.1* |
| *2022-02-08* | *6039* | *USC Path.&Lab.Med.* | *Omicron BA.1* | *Omicron BA.1* |
| *2022-02-08* | *6042* | *USC Path.&Lab.Med.* | *Omicron BA.1* | *Omicron BA.1* |
| *2022-02-08* | *6043* | *USC Path.&Lab.Med.* | *Omicron BA.1* | *Omicron BA.1* |
| *2022-02-08* | *6046* | *USC Path.&Lab.Med.* | *Omicron BA.1* | *Omicron BA.1* |
| *2022-02-09* | *6051* | *USC Path.&Lab.Med.* | *Omicron BA.1* | *Omicron BA.1* |
| *2022-02-09* | *6057* | *USC Path.&Lab.Med.* | *Omicron BA.1* | *Omicron BA.1* |
| *2022-02-11* | *6068* | *USC Path.&Lab.Med.* | *Omicron BA.1* | *Omicron BA.1* |
| *2022-02-12* | *6071* | *USC Path.&Lab.Med.* | *Omicron BA.1* | *Omicron BA.1* |
| *2022-02-14* | *6076* | *USC Path.&Lab.Med.* | *Omicron BA.1* | *Omicron BA.1* |
| *2022-02-14* | *6078* | *USC Path.&Lab.Med.* | *Omicron BA.1* | *Omicron BA.1* |
| *2022-02-15* | *6081* | *USC Path.&Lab.Med.* | *Omicron BA.1* | *Omicron BA.1* |
| *2022-02-15* | *6086* | *USC Path.&Lab.Med.* | *Omicron BA.1* | *Omicron BA.1* |

***Supp. Table 7.*** *Summary of Samples tested by ddPCR and confirmed by WGS*


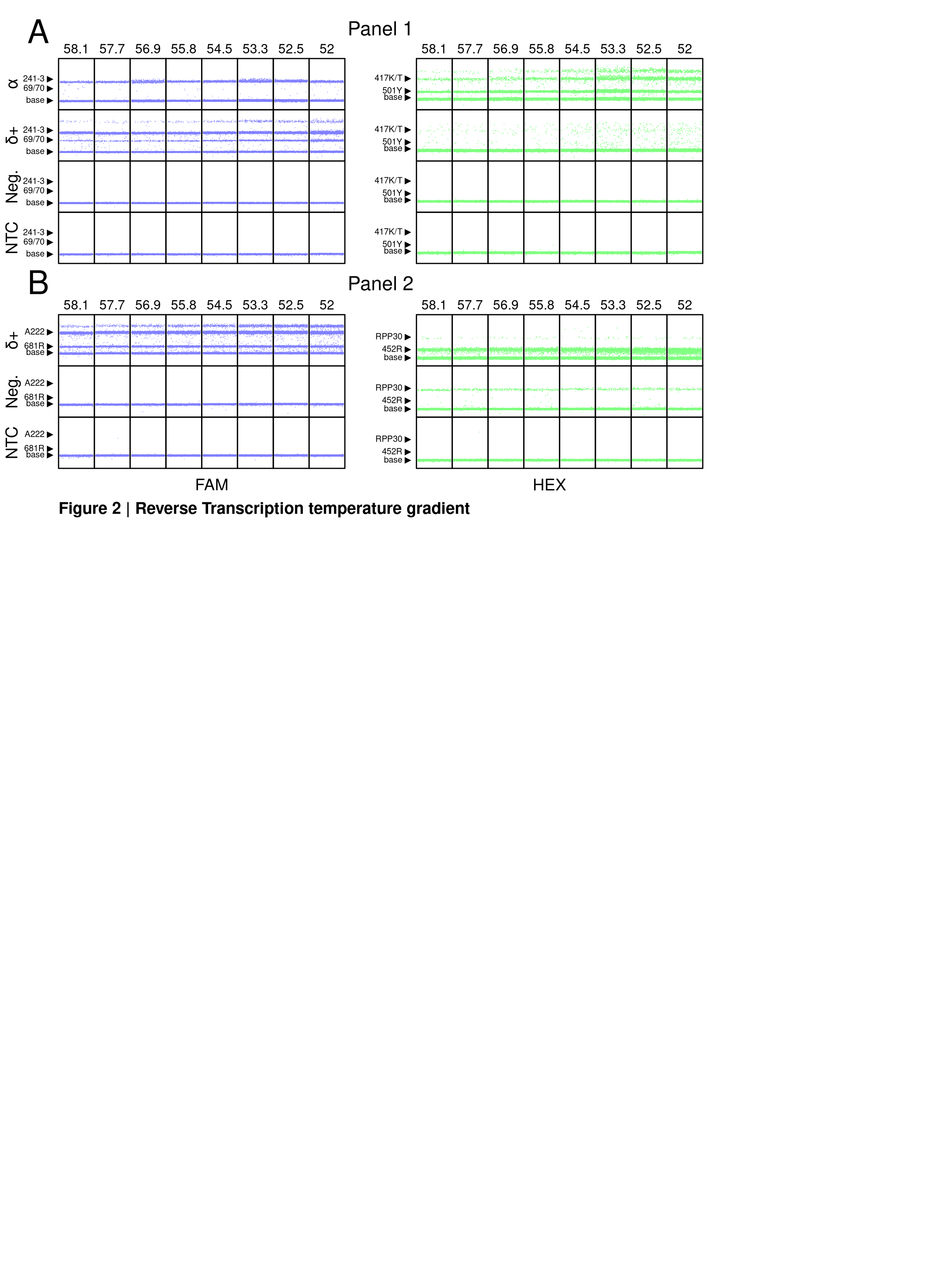


***Supp. Figure 1: Identification of the optimal temperature for the Reverse Transcription step.*** *Negative saliva, no-template control (NTC), and sequenced samples for Alpha and Delta plus control (covering all the assay target) were selected to identify the optimal temperature for the reverse transcription step. Samples were analyzed for panel 1 (A) and 2 (B) in the FAM (blue) and HEX (green) channel with a temperature gradient at the RT step ranging from 58.1 to 52℃. In both panels, 54.5℃ appears to be the highest temperature with optimal amplification.*

**SUPPLEMENTARY FIGURES**


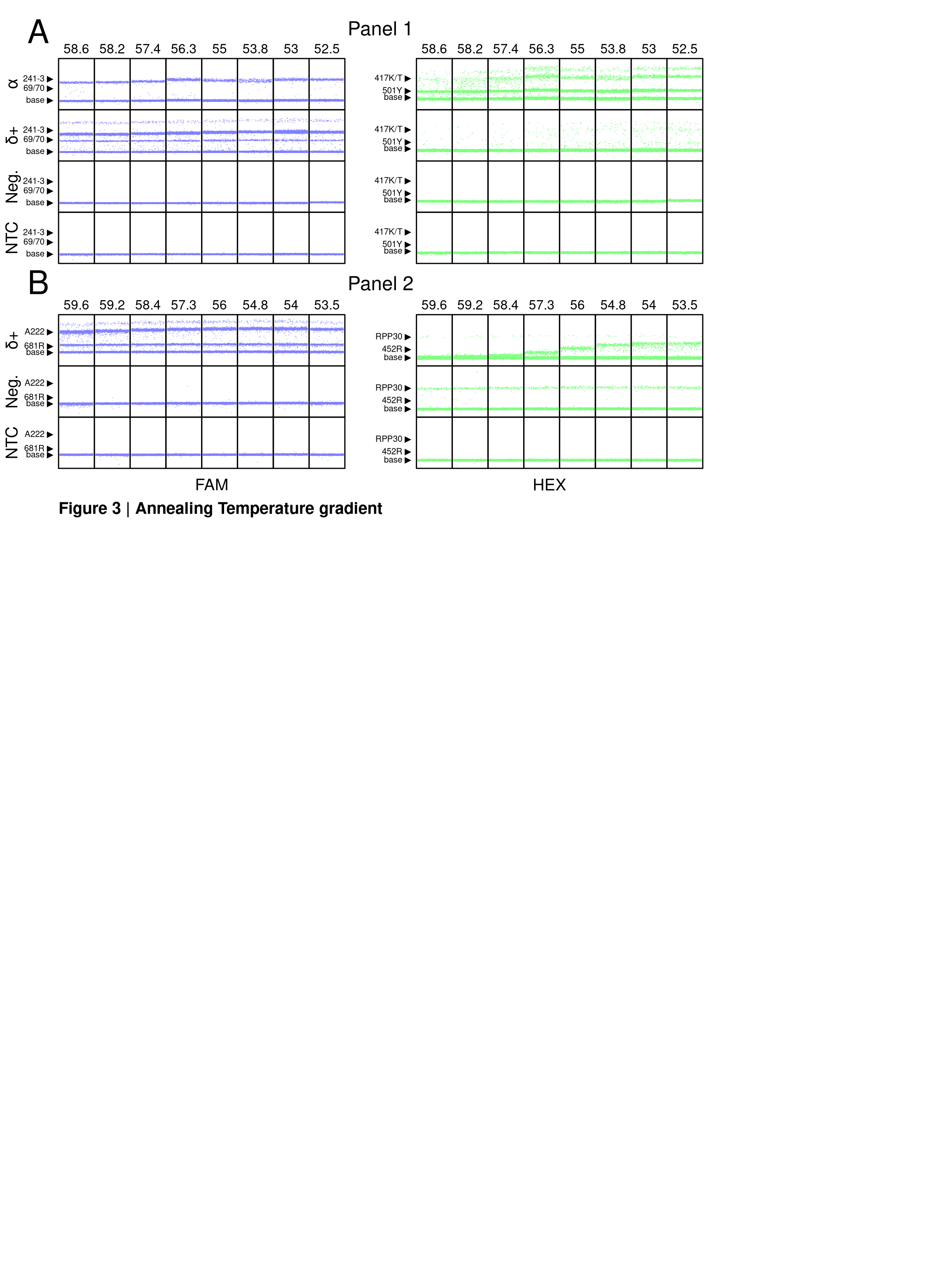


***Supp. Figure 2 : Identification of the optimal temperature for the Annealing step.*** *Negative saliva, no-template control (NTC), and sequenced samples for Alpha and Delta + control were selected to identify the optimal temperature for the annealing step. Samples were analyzed for panel 1 (A) and 2 (B) in the FAM (blue) and HEX (green) channel with a gradient of temperature at the annealing step ranging from 59.6 to 53.5℃. 55℃ (panel 1) and 56℃ (panel 2) appears to be the highest temperatures with optimal amplification for panel 1 and 2, respectively.*


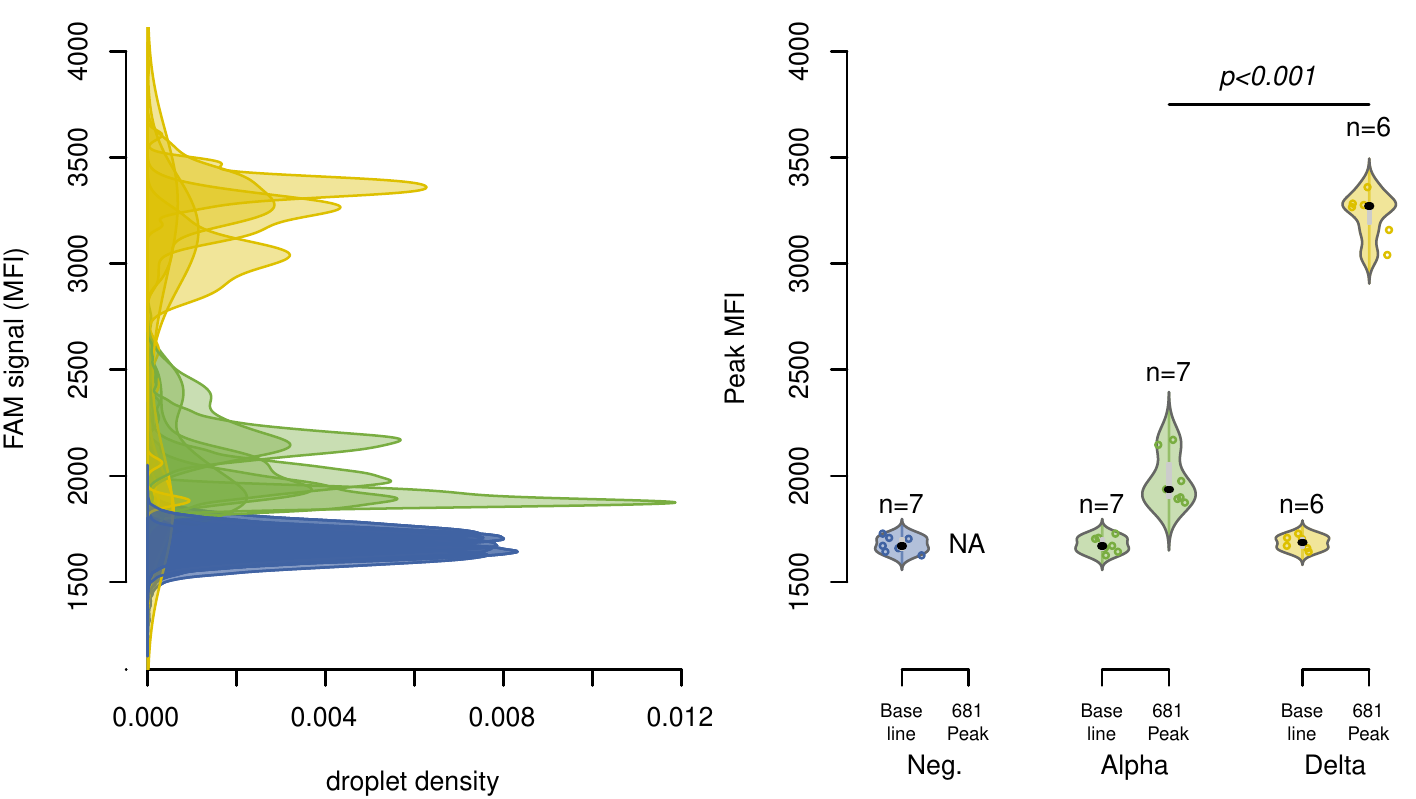


***Supp. Figure 3: Unique amplification pattern for P681R-T715 allows identification of Alpha variant.*** *Replicates of Alpha (green, n=7) and Delta (yellow, n=6) samples, as well as a negative control (blue, n=7) were analyzed by ddPCR on panel. For each replicate, baseline, and peak MFI for this target (in the FAM channel) were identified (left). Results show that signal peaks for the Alpha variant (P681H/T715I) are significantly different from the Delta variant (P681R/T715wt) (right) using a two sided t-test.*


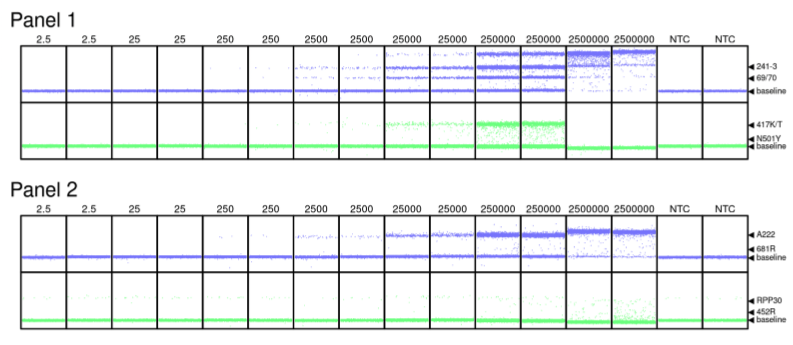


***Supp. Figure 4 – Limit of identification:*** *WA isolate standard sample was used to generate a serial dilution scale ranging from 2.5 to 2,500,000 copies per reaction. Dilutions were run in duplicate for panel 1 (top) and 2 (bottom). Droplet detection allowing clear identification was possible above 250 copies per reaction.*


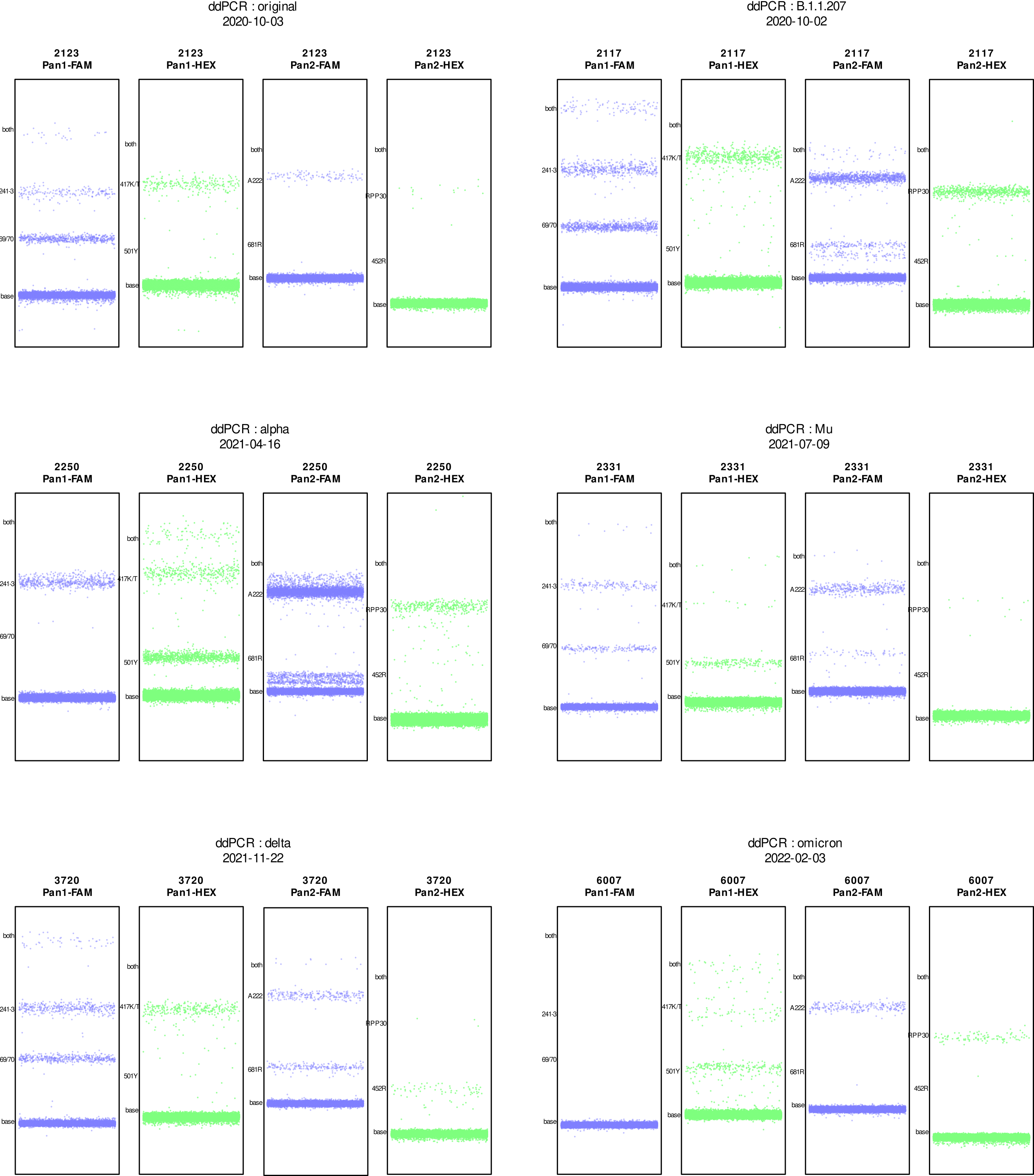


***Supp. Figure 5 – Examples of SARS-CoV-2 variants identified by ddPCR in saliva samples.***
